# Large-scale mapping of mutations affecting zebrafish development

**DOI:** 10.1186/1471-2164-8-11

**Published:** 2007-01-09

**Authors:** Robert Geisler, Gerd-Jörg Rauch, Silke Geiger-Rudolph, Andrea Albrecht, Frauke van Bebber, Andrea Berger, Elisabeth Busch-Nentwich, Ralf Dahm, Marcus PS Dekens, Christopher Dooley, Alexandra F Elli, Ines Gehring, Horst Geiger, Maria Geisler, Stefanie Glaser, Scott Holley, Matthias Huber, Andy Kerr, Anette Kirn, Martina Knirsch, Martina Konantz, Axel M Küchler, Florian Maderspacher, Stephan C Neuhauss, Teresa Nicolson, Elke A Ober, Elke Praeg, Russell Ray, Brit Rentzsch, Jens M Rick, Eva Rief, Heike E Schauerte, Carsten P Schepp, Ulrike Schönberger, Helia B Schonthaler, Christoph Seiler, Samuel Sidi, Christian Söllner, Anja Wehner, Christian Weiler, Christiane Nüsslein-Volhard

**Affiliations:** 1 Department 3 – Genetics, Max-Planck-Institut für Entwicklungsbiologie, Spemannstr. 35/III, 72076 Tübingen, Germany; 2 Department of Internal Medicine III – Cardiology, University of Heidelberg, Im Neuenheimer Feld 350, 69120 Heidelberg, Germany; 3 Max Planck Institute for Molecular Genetics, Ihnestr. 63-73, 14195 Berlin, Germany; 4 Laboratory for Alzheimer's and Parkinson's Disease Research, Adolf-Butenandt-Institute, Department of Biochemistry, LMU, Schillerstr. 44, 80336 München, Germany; 5 Team 31 – Vertebrate Development and Genetics, Wellcome Trust Sanger Institute, Wellcome Trust Genome Campus Hinxton, Cambridge, CB10 1SA, UK; 6 Center for Brain Research – Division of Neuronal Cell Biology, Medical University of Vienna, Spitalgasse 4, 1090 Vienna, Austria; 7 Centre for Cellular and Molecular Dynamics, Department of Anatomy and Developmental Biology, University College London, Gower St., London WC1E 6BT, UK; 8 3. Physikalisches Institut, Universität Stuttgart, Pfaffenwaldring 57, 70569 Stuttgart, Germany; 9 Department of Molecular, Cellular and Developmental Biology, Yale University, P.O. Box 208103, New Haven, CT 06520-8103, USA; 10 Institut für Klinische Pharmakologie und Toxikologie, Charité – Universitätsmedizin Berlin, Campus Benjamin Franklin, Hindenburgdamm 30, 12200 Berlin, Germany; 11 NMI – Natural and Medical Science Institute at the University of Tübingen, Markwiesenstr. 55, 72770 Reutlingen, Germany; 12 Institute of Physiology Dept. II and Tübingen Hearing Research Centre THRC, University of Tübingen, Elfriede-Aulhorn-Str. 5, 72076 Tübingen, Germany; 13 Institute of Pathology, Rikshospitalet, Sognsvannveien 20, 0027 Oslo, Norway; 14 Current Biology, Elsevier London, 84 Theobald's Rd., London WC1X 8RR, UK; 15 Institute of Zoology, University of Zurich, Winterthurerstr. 190, 8057 Zürich, Switzerland; 16 Oregon Hearing Research Center and Vollum Institute, Oregon Health & Science University, 3181 SW Sam Jackson Pk. Rd., Portland, OR 97239, USA; 17 Division of Developmental Biology, National Institute for Medical Research, The Ridgeway, Mill Hill, London NW7 1AA, UK; 18 Laboratory for Magnetic Brain Stimulation, Behavioral Neurology Unit, Beth Israel Deaconess Medical Center, Harvard Medical School, 330 Brookline Ave., Boston, MA 02215, USA; 19 Howard Hughes Medical Institute, University of Utah, 15 North 2030 East, Salt Lake City, UT 84112, USA; 20 MDC – Max-Delbrück-Centrum für Molekulare Medizin, Berlin-Buch, Robert-Rössle-Str. 10, 13092 Berlin, Germany; 21 Cellzome AG, Meyerhofstr. 1, 69117 Heidelberg, Germany; 22 Ingenium Pharmaceuticals AG, Fraunhoferstr. 13, 82152 Martinsried, Germany; 23 Abt. Kinderheilkunde I, Children's Hospital, University of Tübingen, Hoppe-Seyler-Str. 1, 72076 Tübingen, Germany; 24 IMP – Research Institute of Molecular Pathology, Dr. Bohr-Gasse 7, 1030 Vienna, Austria; 25 Department of Medicine, University of Pennsylvania School of Medicine, 1230 Biomedical Research Building II/III, 421 Curie Blvd., Philadelphia, PA 19104, USA; 26 Department of Pediatric Oncology, Dana-Farber Cancer Institute, Harvard Medical School, Mayer Building 630, 44 Binney St., Boston, MA 02115, USA; 27 Team 30 – Vertebrate functional proteomics laboratory, Wellcome Trust Sanger Institute, Wellcome Trust Genome Campus Hinxton, Cambridge, CB10 1SA, UK; 28 Department of Cell Biology, Max-Planck-Institute of Biochemistry, Am Klopferspitz 18, 82152 Martinsried, Germany

## Abstract

**Background:**

Large-scale mutagenesis screens in the zebrafish employing the mutagen ENU have isolated several hundred mutant loci that represent putative developmental control genes. In order to realize the potential of such screens, systematic genetic mapping of the mutations is necessary. Here we report on a large-scale effort to map the mutations generated in mutagenesis screening at the Max Planck Institute for Developmental Biology by genome scanning with microsatellite markers.

**Results:**

We have selected a set of microsatellite markers and developed methods and scoring criteria suitable for efficient, high-throughput genome scanning. We have used these methods to successfully obtain a rough map position for 319 mutant loci from the Tübingen I mutagenesis screen and subsequent screening of the mutant collection. For 277 of these the corresponding gene is not yet identified. Mapping was successful for 80 % of the tested loci. By comparing 21 mutation and gene positions of cloned mutations we have validated the correctness of our linkage group assignments and estimated the standard error of our map positions to be approximately 6 cM.

**Conclusion:**

By obtaining rough map positions for over 300 zebrafish loci with developmental phenotypes, we have generated a dataset that will be useful not only for cloning of the affected genes, but also to suggest allelism of mutations with similar phenotypes that will be identified in future screens. Furthermore this work validates the usefulness of our methodology for rapid, systematic and inexpensive microsatellite mapping of zebrafish mutations.

## Background

Large-scale mutagenesis screens in the zebrafish employing the mutagen ENU have isolated several hundred mutant loci that represent putative developmental control genes [1,2]. In order to realize the potential of such screens, systematic genetic mapping of the mutations is necessary. Genome scanning by bulked segregant analysis with microsatellite markers is the method of choice for such purposes, as a rough map position can be quickly obtained [3,4]. In the zebrafish it is easy to perform mapcrosses against a polymorphic reference line, followed by brother-sister matings among the F1 generation. Linkage to a microsatellite marker can then be found by comparing the band intensities of marker alleles in a pool of mutant F2 individuals with a pool of their wildtype siblings. Because full sibships are analyzed the genetic distance between the mutant locus and a microsatellite can be determined by a simple count of recombinations.

The established reference map for the zebrafish genome is the MGH map [5-7] which was generated by scoring 3,881 microsatellite markers (all of them CA repeats) on a panel of 48 diploid F2 fish of an India × AB reference cross. It covers 2,295 centimorgans (cM) at a resolution of 1.2 cM. Because the MGH markers do not necessarily show a usable polymorphism in reference crosses of Tü × WIK our first task was to identify markers that could be used in such a cross.

## Results and discussion

### Selection of markers for genome scanning

Two sets of microsatellite markers for scanning the genome were developed in parallel with the mutant mapping effort. The starting point was the testing of 314 markers for polymorphism in Tü × WIK crosses [8]. 72 markers (3 per chromosome) were selected that showed a polymorphism between Tü and WIK, with bands easily distinguishable on agarose gels, in at least three out of five reference crosses (zebrafish genome scan set version 1, or G1). Additional markers from the MGH map that had shown a robust polymorphism in fine-mapping experiments were subsequently added, while markers that never gave any confirmed linkage in our experiments or that were omitted from the MGH map were removed from our set, eventually resulting in the G4 set of 192 markers [9]. An alternate set of markers was generated by testing another 1,092 microsatellite markers from the MGH map in five reference crosses. 178 of these markers were polymorphic in all five reference crosses. Together with 14 additional markers these were selected for the H2 set of 192 markers (Table 1).

The average distance between markers of the G4 set is 11.6 cM, and all distances are smaller than 36 cM, except for a 71.1 cM interval on LG21 (between Z4425 and Z1497). Within this particular interval few MGH markers are available, and no suitably polymorphic marker could be identified in our reference crosses. For the H2 set the average distance is 11.5 cM, and all distances are smaller than 53.8 cM, except for a 83.3 cM interval on LG21. The more uneven chromosomal distribution of markers in the H2 set reflects the fact that frequently the best markers available were already used in the G4 set.

Our mapping methodology as described below can theoretically detect significant linkage over a distance of approximately 36 cM (assuming the genotyping of 48 mutant individuals). However, since the LOD score is proportional to the number of individuals scored, this range can be easily increased by adding more mutant individuals if a linkage is questionable. Our marker sets therefore cover the genome adequately to detect significant linkage with the great majority of mutant loci. All the mutant loci mapped in this work have confirmed linkage to at least one G4 or H2 marker (not shown if the closest flanking markers were selected from outside the sets).

### Mapping of mutant loci

We report here on the mapping of 319 mutant loci identified in the ENU-based Tübingen I mutagenesis screen [1,2] and subsequent screening among the mutant collection (Additional file 1). For 42 of the loci the corresponding genes have already been identified by other researchers, as listed by the ZFIN database [10]; they are included as controls for our mapping procedure (see below). Not included are 70 successfully mapped loci for which the corresponding genes were already published by ourselves or such a publication is in preparation, or the carriers of which were lost after mapping.

For each mutation we crossed mutant carriers against the polymorphic reference line WIK which was established in our lab for this purpose [8]. Brother-sister matings were performed in the F1 and the F2 progeny was sorted by phenotype. DNA was prepared on 96-well plates, and aliquots of 36 – 48 mutant F2 individuals and their wildtype siblings were pooled. Genome scanning was performed by PCR of the mutant and sibling pools with the markers of the G4 marker set, and the band intensities on agarose gels were quantified semi-automatically using NIH Image software as well as visually assessed to identify potential linkages. Mutant and sibling pools representing up to 24 different mutations were tested in parallel. Verification of the best potential linkages (up to six) for each mutation was then attempted by performing PCR of the respective marker with the individual mutants and siblings that had been used for pooling, and counting the recombinant genotypes (for the genotype data see Additional file 2). Siblings were always included on the same gel as a control to confirm that the marker is polymorphic and the two polymorphic bands appear at the proper frequency. If no potential linkage could be verified for a mutation and sufficient material was available, the procedure was repeated once with the G4 marker set, and another two times with the H2 marker set. If possible, DNA was prepared from a different F1 pair for each genome scan, since the Tübingen and WIK lines used are not isogenic and markers that show no usable polymorphism in progeny of one F2 pair are therefore sometimes usable in progeny of another one.

A potential linkage was considered confirmed if it had a two-point LOD score equal or greater than 3. The individuals were then genotyped for all polymorphic markers from the same marker set and chromosomal region in order to identify, if possible, a pair of markers flanking the mutation, and if that was not possible, the two closest markers on one side of the mutation. Occasionally additional markers not in the chosen marker set were also included in the genotyping. Decisions on whether or not a mutation was flanked by two markers were based on whether recombinations with the markers occurred independently. For details of the mapping procedure and the calculation of map positions see the Methods section and [9].

In total, mapping was attempted for 486 mutations from the Tübingen I screen and subsequent screens of the mutant collection and successful for 389, giving a success rate of 80 %. 12 of these could be mapped only with the H2 set. Unsuccessful mapping experiments were due to difficulties in obtaining sufficient F2 individuals and to PCR problems as well as to a lack of polymorphic markers in our marker set. Among the mutations to be mapped, a group of 63 was prioritized based on interest in their phenotypes. For each of these several additional mapcrosses were set up (data not shown). 56 mutations of this group, or 89 % were successfully mapped, providing a lower limit for the percentage of mutations that our marker sets and methodology is capable of mapping if sufficient F2 individuals are available. The biggest distance to markers on either side at which we could confirm linkage was 31.9 cM (for the mutation *spt*), approaching the theoretical cutoff of 36 cM.

### Chromosomal distribution of mutant loci

Between 1,400 and 2,400 zebrafish genes have been estimated to have visible mutant phenotypes in embryonic and early larval development [1,11]. Therefore the loci reported in this work represent at least one eighth and possibly as much as quarter of all the loci that can be mutated to give a visible phenotype.

**Figure 1:**
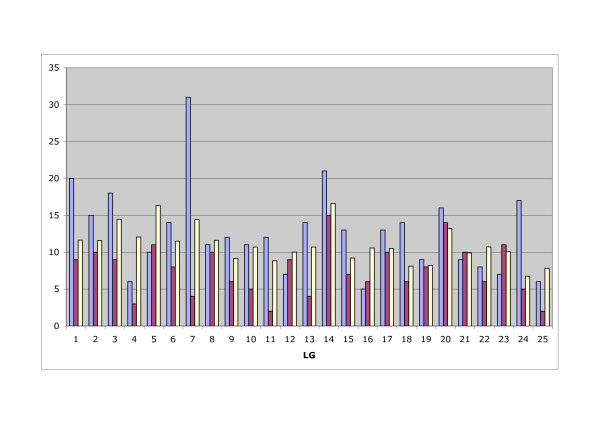
**Distribution of mapped mutations among the zebrafish chromosomes**. Light blue, ENU mutations mapped in the present work. Purple, insertional mutations from the laboratory of N. Hopkins ([11][12] and unpublished data, available from ZFIN [10]), shown for comparison. Numbers for insertional mutations were obtained by searching ZFIN for mutations with a "hi" designation assigned to each linkage group and eliminating multiple hits of the same gene as well as mutations with ambiguous chromosomal assignments. Yellow, Ensembl genes predictions for each chromosome (× 100) (Ensembl release Zv6, available from Ensembl [13]) The number of mapped mutations or genes is indicated on the vertical axis, the linkage group (LG) number on the horizontal axis.

The number of mapped loci assigned to each chromosome is between 6 and 32 (on average 12.8 ± 5.8) (Figure 1). These numbers are not significantly correlated with the number of mutant loci per chromosome identified by insertional mutagenesis in the laboratory of N. Hopkins ([11,12] and unpublished data, available from ZFIN [10]) (R^2 ^= 0.02 assuming a linear regression relationship) or with the number of Ensembl genes per chromosome in the Ensembl Zv6 assembly [13] (R^2 ^= 0.19); by comparison, the values of Amsterdam et al. have a slightly stronger correlation to the number of Ensembl transcripts (R^2 ^= 0.28). Because mapping with our methodology was successful for 80% of all mutations for which it was attempted, possible deficiencies of the mapping method cannot fully account for this low correlation. Rather, it probably reflects an uneven distribution of genes with specific, visible phenotypes in embryos or early larvae as identified in ENU mutagenesis screening, and the absence of such selectivity in the insertional mutagenesis experiment, demonstrating that both types of mutagenesis experiments complement each other in their coverage of their genome. Moreover, we cannot rule out region-specific differences in ENU mutagenesis efficiency.

### Assessment of mapping quality

In order to assess the quality of our mapping data we looked at the 42 mutant loci that were cloned by other researchers. For 21 of these independently derived map positions of the affected gene are publicly available on ZMAP (an integrated map produced by intercalating data from several mapping panels into the MGH genetic map, available from ZFIN [10]; Allen Day, Tom Conlin and John H. Postlethwait, unpublished) (Table 2).

A comparison of the linkage group assignments shows that two of the 21 genes (*frs*/*slc25a *and *ovl*/*ift88*) are assigned to a different linkage group by ZMAP, in both cases based on results from the Heat Shock (HS) panel [14-16]. However, several published linkages to genetic markers support our linkage group assignment of *frs*/*slc25a *[17] while our assignment of *ovl/ift88 *is supported by the T51 panel (as shown on the ZFIN website) and by the latest version of the HS map [18]. In conclusion, none of our linkage group assignments is conclusively contradicted by gene mapping.

Next we compared the map positions of the mutations with those of the genes on ZMAP (using the median of the ZMAP positions if a gene was placed on more than one mapping panel). If we assume the gene positions to be correct, we obtain a standard error of our mutant map positions of 6.1 cM. Further assuming a normal distribution of errors, we can predict that approximately 95 % of the genes should be within 12.2 cM (two standard errors) of the rough mapping position of the mutation. Indeed, 17 out of the 19 genes mapped on the same chromosome (90 %) are within two standard errors of the mutation, and 16 out of 19 (84 %) within one standard error. Actually both mutation and gene mapping contribute to the observed errors to an unknown degree, so that 6.1 cM merely represents an upper limit for the standard error of our mapping procedure.

## Conclusion

We have obtained rough map positions for over 300 zebrafish mutants with an accuracy of approximately 6 cM and thereby validated the usefulness of our methodology for rapid, systematic and inexpensive microsatellite mapping of zebrafish mutations. The dataset that we have produced is a first step towards identification of the genes affected by the 277 mutations that are not yet cloned.

In candidate gene approaches, our data can substantially narrow down the number of candidate genes, since on the order of 99 % of the genome are outside the two-standard-errors confidence limit of our map positions. Positional cloning approaches in the absence of obvious candidate genes will still require fine mapping by genotyping of additional individuals and identification of more closely linked markers, using the flanking markers identified by us as starting points. Particularly thorough fine-mapping is required in centromeric regions because the genetic recombination rate is often several-fold reduced in such regions [19], an effect that can be easily observed in the zebrafish by comparing the genetic map and the radiation hybrid map [9]. Nevertheless, we expect our map positions to be useful even without knowledge of the affected genes, as they can suggest allelism of mutations with a similar phenotype identified in future screens.

We have found that a relatively small number of microsatellite markers is sufficient to scan almost the entire genome and that the experimental procedures are robust and easy to perform. Other methods that have been proposed for the mapping of mutant loci in the zebrafish include half-tetrad analysis with microsatellite markers, genome scanning with SNPs and microarray based SNP mapping. While half-tetrad analysis requires only 25 markers to obtain a linkage group assignment [20-22], it has the disadvantage that gynogenetic diploid fish must be generated first, which makes this approach less convenient for high-throughput analysis. In the course of the ongoing zebrafish genome project, more than 50,000 SNPs have been identified [23] offering an enticing alternative to microsatellite markers, but SNP genotyping is far more costly than the agarose based method employed by us. Genotyping of SNPs in a bulked segregant panel is also possible by microarray hybridization [24]. However, the SNPs identified to date are specific to the strains they were developed from and may not be informative in mapcrosses performed with different strains (such as ours). Furthermore such a microarray experiment replaces only two steps in our mapping procedure, namely the pooled PCR and its associated gel run, which represent only a minor part of the total mapping effort, as compared to fish breeding, sorting of F2 embryos and confirmation of the bulked segregant results by genotyping of F2 individuals. Future microarray based approaches may make it possible to dispense with the genotyping of individuals entirely, provided that a very large number of SNPs can be multiplexed in a single microarray hybridization such that it immediately provides a reliable map position. Meanwhile, genome scanning with microsatellite markers remains the method of choice as it is equally suitable for the mapping of individual mutations by laboratories with limited genomics resources, and for high throughput projects such as ours.

## Methods

### Fish breeding

Mapcrosses were set up between mutant carriers and the laboratory reference line WIK [8] and brother-sister matings were performed between F1 individuals following standard laboratory procedures [25].

### DNA preparation

F2 embryos were sorted by phenotype and stored in Eppendorf tubes with 100 % MeOH at -70°C until use. Single embryos were arrayed on a 96-well microtiter plate with a glass Pasteur pipette. The MeOH was evaporated on a PCR block at 70°C and 25 μl of 1.7 mg/ml Proteinase K in 1 × TE was added to each well. The plate was covered with sealing film and heated to either 55°C or 70°C for 240 min and to 94°C for 10 min in a thermocycler. 10 μl of each of the sibling and mutant lysates was pooled and 45 μl sterile ddH_2_O was added to the remainder. Plates were stored at -20°C.

### Genotyping

PCR was initially performed on mutant and sibling pools for genome scanning, and subsequently on the individuals that had been used for the pooling in order to confirm potential linkages to specific markers. 20 μl PCR reactions were set up from 14.28 μl of reaction mix (2 μl of 10 × PCR buffer, 0.04 μl each of 100 mM dATP, dCTP, dGTP and dTTP, 12.12 μl water), 0.16 μl each of 20 mM forward and reverse primer, 0.4 μl of 5U/μl Taq polymerase, and 5 μl of template DNA. 10 × PCR buffer contained 100 mM Tris-HCl (pH 8.3), 500 mM KCl, 15 mM MgCl_2 _and 0.1 % (w/v) gelatin. All pipetting was done with a Biomek 2000 robot. Cycling was carried out by initially denaturating at 94°C for 2 min, 35 cycles of denaturation at 94°C for 30 sec, annealing at 60°C for 30 sec and extension at 73°C for 1 min, and a final extension at 73°C for 5 min. 5 μl of 6 × loading buffer were added to each sample, and electrophoresis was carried out at 200 V for 45 min in 1 × TBE buffer, on 2 % agarose gels. Gels were imaged and scored semi-automatically with NIH Image and a set of custom-designed macros.

### Calculation of map positions

Distances between mutations and markers were calculated by determining the recombination fraction in the mutant F2 individuals and applying the Kosambi mapping function. Linkages with a two-point LOD score equal or greater 3 were regarded as significant.

In order to place a mutation in the genetic interval between the closest marker and another linked marker we determined whether recombinations for both of them were correlated. For this purpose we considered only single recombinants for the closest marker, i.e. heterozygotes. If the majority of these were heterozygous for the second marker we regarded the recombinations as uncorrelated and placed the mutation in the interval between the markers. Otherwise we placed the mutation outside the interval in the direction opposite from the second marker.

Assuming complete meiotic interference, i.e. only a single recombination event per chromosome, all recombinants for the first marker should be either non-recombinant for the second marker if the markers flank the mutation, or heterozygous if both markers are on the same side of the mutation. In our data approximately half of the mutations gave results in between these extremes. This may be due to occasional contaminations of the PCR assays but also to less than complete meiotic interference, which would allow a second recombination in the same individual. We therefore did not eliminate any contradictory individuals from the calculation of genetic distances as they may represent a genuine second recombination.

If a mutation could be placed in an interval between two markers, a map position was calculated by scaling the observed distances between the mutation and the markers so as to fit into the published distance between the markers. In the remaining cases only the distance to the closest marker was used to calculate the map position. A FileMaker Pro 5 database was used to store the scoring data and perform the calculations [9]. The latest version of the MGH map, available through ZFIN [10], was used as a reference for calculating map positions.

## Authors' contributions

RG implemented the mapping approach, supervised the project, analysed the results and drafted the manuscript. CNV initiated and supported the project. The remaining authors contributed equally to fish breeding and sorting by phenotype, PCR reactions and electrophoresis and scoring of gel images. GJR, BR and ER also evaluated the polymorphism of microsatellite markers for the selection of marker sets. All authors read and approved the final manuscript.

### Note added in proof

For the following mutations, still listed as uncloned in Additional file 1, the corresponding genes have been reported by other researchers: beo, blu, hap, leo, obe, san, stu. For references see the ZFIN database  [10].

## Supplementary Material

Additional file 1 **Mapping results**. Listed are 319 mutant loci identified in the ENU-based Tübingen I mutagenesis screen [1,2] and subsequent screening among the mutant collection. Descriptions of all listed mutations are available from the ZFIN database [10]. The list includes 42 mutations for which the corresponding gene was identified by other researchers according to ZFIN. References to the original publications are not included due to space constraints, but are likewise available from ZFIN. We recognize the priority of mapping results already published for these 42 mutations, but include the mutations here as controls for our mapping data (see Table 2). Allele: allele name. Abbr: mutant abbreviation (or "*unm*" for unnamed mutant). LG: linkage group. Pos: map position deduced from the two markers listed further to the right, in cM from the top of the linkage group. Gene: symbol of the corresponding gene as listed by ZFIN (or "n.d." if not identified). Marker1: closest marker. Marker2: closest marker on the other side of the mutation (if available) or second closest marker on the same side. Pos1, Pos2: marker position on the MGH map, in cM from the top of the linkage group. Dis1, Dis2: distance between mutant locus and marker, in cM. LOD1, LOD2: LOD score for two-point linkage between the mutant locus and marker ("inf" for infinite if no recombinants were found). n1, n2: number of F2 individuals successfully scored. * Marker 2 was determined to be on the same side of the mutant locus as marker 1, since the majority of recombinants for marker 1 is also recombinant for marker 2. However, no usable marker was found on the other side. In these cases only the distance from marker 1 is used to calculate the map position. ** One of the F1 individuals is homozygous for marker 2, allowing only recombinations in the other individual to be scored. Such linkages are listed in order to support chromosomal assignments if no other usable marker was found. However, the distances are not comparable to the sex-averaged map because male and female recombination rates differ to an unknown extent. In these cases the mutation is placed at the position of marker 1. *** *ali *and *bxe *have ambiguous positions because marker 1 and marker 2 are on the same side of the mutant locus and the distance to both markers is the same. Thus, *ali *can be placed either at 107.5 or 134.0 cM from the top of LG21, and *bxe *either 34.7 or 78.7 cM from the top of LG13. Click here for file

Additional file 2 **Genotypes**. This file lists the scoring results of individual F2 fish that were used to determine the map positions of the mutations. Row: template plate row, identifies the individuals scored; one row of the file corresponds to two rows of wells on a microtiter plate. Abbr: mutant abbreviation. Allele: mutant allele. Marker: marker name. Genotypes: 24 genotypes, encoded as follows: "1", homozygous for the upper band (which may be either Tü or WIK, dependent on the marker and F1 cross), "2", homozygous for the lower band, "3", heterozygous, "0" or "-", not determined (bad gel lane or microtiter well, respectively). Click here for file

## Figures and Tables

**Table 1:** Sets of microsatellite markers used for scanning the zebrafish genome

**LG**	**Marker set G4**	**Marker set H2**
1	Z4593, Z9394, Z5508, Z1705, Z1351, Z9704, Z11464, Z6802, Z1781	Z11913, Z6384, Z9977, Z6415, Z7287, Z9395, Z22319, Z10978, Z11618
2	Z7634, Z4662, Z3430, Z1406, Z6617, Z1703, Z20550	Z13620, Z7361, Z9361, Z13281, Z21490, Z7678, Z10302, Z22544
3	Z872, Z8208, Z15457, Z9964, Z11227, Z3725, Z20058, Z6019	Z8364, Z9843, Z7419, Z8681, Z9662
4	Z1525, Z9920, Z21636, Z7490, Z984	Z7629, Z10983, Z23058, Z9319, Z17278, Z6503, Z11566
5	Z15414, Z11496, Z6727, Z10456, Z1390, Z3804, Z14143, Z4299, Z1202	Z6916, Z9106, Z9109, Z9969, Z7313, Z7291, Z22523, Z21290, Z10484, Z7318, Z13304
6	Z740, Z13275, Z880, Z6624, Z10183, Z5294, Z13614, Z7666, Z4297, Z1680	Z15448, Z8447, Z22253, Z13328, Z8245, Z9254, Z6330, Z9652, Z17248, Z9230, Z21901
7	Z3273, Z10785, Z1206, Z4706, Z1182, Z1059, Z8156, Z1239, Z13880, Z13936, Z5563	Z7479, Z7555, Z7069, Z8975, Z8540, Z6273, Z10451, Z22628
8	Z1634, Z1068, Z4323, Z13412, Z21115, Z789, Z10929, Z3526	Z7962, Z11492, Z21483, Z10121, Z11946, Z23039, Z23009
9	Z1777, Z6268, Z4673, Z5080, Z1805, Z20031, Z10789, Z4577	Z22173, Z9923, Z6845, Z9439, Z6574, Z6336, Z9975
10	Z9199, Z6410, Z8146, Z13632, Z1145, Z9701, Z3260	Z6648, Z22661, Z8318, Z9574, Z8705, Z7558, Z15444
11	Z10919, Z3362, Z13411, Z1393, Z3527, Z1590	Z11865, Z7657, Z11067
12	Z1778, Z21911, Z1473, Z4188, Z1358	Z7409, Z8755, Z8460, Z10225, Z22666, Z6442, Z7834, Z20142, Z11903
13	Z1531, Z5643, Z6104, Z13611, Z5395, Z1627, Z7102, Z6657, Z1826, Z6007	Z10513, Z9049, Z11695, Z22022, Z20208, Z20379, Z9357, Z8617, Z11459
14	Z1523, Z5436, Z1536, Z5435, Z4203, Z22107, Z1226, Z3984, Z1801	Z6545, Z8471, Z11694, Z9720, Z4592, Z20663, Z20214, Z9789, Z11837
15	Z6312, Z6712, Z21982, Z4396, Z11320, Z13230, Z13822, Z7381, Z5223	Z20627, Z21452, Z22027, Z11323, Z7070, Z6024, Z13927
16	Z3741, Z21155, Z6365, Z10036, Z1215, Z4670	Z10217, Z10671, Z11452, Z20177, Z6329, Z6240, Z6293, Z7956, Z20704
17	Z4268, Z1490, Z22083, Z22674, Z9847, Z1408, Z4053	Z7625, Z9179, Z8862, Z9633, Z9830, Z13631, Z13643
18	Z1136, Z1144, Z13329, Z8488, Z10008, Z3558, Z9154, Z5321	Z14136, Z9484, Z8525, Z7142, Z13260, Z14011
19	Z4009, Z160, Z3782, Z3816, Z11403, Z6661, Z7926, Z1803	Z1544, Z22649, Z22818, Z13773, Z6079, Z13727, Z9050, Z7265, Z10273
20	Z9334, Z10056, Z11841, Z3964, Z7158, Z3954, Z22041, Z8554, Z4329	Z10177, Z17204, Z10901, Z10756, Z7568, Z6973
21	Z3476, Z1274, Z4492, Z10960, Z4425, Z1497, Z4074	Z6174, Z6243, Z8230, Z9728, Z9236, Z20446, Z6087
22	Z1148, Z10673, Z9402, Z230, Z10321, Z21243	Z6613, Z10028, Z10324, Z11262, Z20168, Z21507, Z21252, Z11679
23	Z8945, Z4003, Z15422, Z4421, Z3157, Z176, Z1773	Z8362, Z11495, Z20643, Z7550, Z11391, Z14008
24	Z5075, Z1584, Z5413, Z23011, Z3399, Z22375, Z5657, Z3901	Z7349, Z10961, Z10458, Z13695, Z10529, Z6438, Z21908, Z6296, Z7132, Z9673
25	GOF15, Z1378, Z3490, Z5669, Z1462	Z21929, Z21722, Z13232, Z15480, Z10010, Z10578, Z21181

The G4 marker set [9] and the newly developed H2 marker set each consist of 192 microsatellite markers from the MGH map [5][6] which we selected for genome scanning in Tü × WIK crosses and electrophoresis on agarose gels. Up to two genome scans per mutation were performed with the G4 set (or its earlier versions). If no linkage could be confirmed and sufficient material was available, another two scans were subsequently performed with the H2 set. Additional markers from the MGH map were occasionally employed for scoring of mutant individuals. LG: linkage group.

**Table 2:** Comparison of mutant and gene positions

**Abbr.**	**Allele**	**LG**	**Pos.**	**Gene**	**Gene Pos.**	**Panels**	**Diff.**
*acc*	*tq206*	3	52.3	*atp2a1*	54.00	T51, HS	-1.70
*boz*	*th211*	15	57.3	*dharma*	57.77	LN54, HS	-0.47
*cdy*	*te216*	11	0.5	*slc11a2*	0.00	HS	0.50
*con*	*tf18b*	20	96.2	*disp1*	98.57	HS	-2.37
*dtr*	*tm276b*	6	85.3	*gli1*	85.40	MGH, HS	-0.10
*eya1*	*tm90b*	24	36.2	*eya1*	42.90	LN54, T51	-6.70
*frs*	*tg280a*	8	82.2	*slc25a37*	[LG17]	HS ^a^	n.d.
*ika*	*tm127c*	14	33.0	*fgf24*	34.97	T51	-1.97
*kgg*	*tl240a*	14	57.2	*cdx4*	56.00	T51, HS, MOP	1.20
*mfn*	*tc263*	1	37.2	*tll1*	37.10	MGH, HS	0.10
*mib*	*ta52b*	2	20.9	*mib*	35.54	T51	-14.64
*mon*	*tg234*	8	42.5	*trim33*	52.85	HS, T51	-10.35
*mot*	*tm303c*	16	41.6	*epb41*	46.90	T51	-5.30
*nic*	*dtbn12*	6	31.1	*chrna1*	43.62	HS, T51, MOP, LN54	-12.52
*oep*	*tz257*	10	20.0	*oep*	12.20	LN54, T51	7.80
*ovl*	*tz288*	9	50.1	*ift88*	[LG13]	HS ^b^	n.d.
*ris*	*tb237*	17	47.9	*sptb*	45.50	HS, MGH	2.40
*spt*	*tm41*	8	94.2	*tbx16*	96.03	T51, LN54, MOP	-1.83
*suc*	*tf216b*	19	20.9	*edn1*	11.90	MGH, LN54, T51	9.00
*ubo*	*tp39*	16	24.2	*prdm1*	28.08	LN54	-3.88
*you*	*ty97*	7	40.5	*scube2*	37.90	HS	2.60

Map positions of mutations that were mapped in our project and cloned by other researchers are shown together with the ZMAP positions of the affected genes (publicly available from ZFIN [10]). The gene positions originate from several mapping panels: MGH [5] [6] [7]; Heat Shock (HS) [14] [15] [16], T51 [26] [27] [28] (Yi Zhou and Leonard Zon, unpublished data); LN54 [29] [30]; and Mother of Pearl (MOP) [31]. References to the original publications of the individual genes are not included here due to space constraints, but are likewise available from ZFIN. The map positions were subsequently intercalated into the MGH genetic map to produce the integrated ZMAP (Allen Day, Tom Conlin and John H. Postlethwait, unpublished). If a gene was mapped on more than one panel, the median position is shown. Only genes or gene-encoded markers directly placed on ZMAP are shown (rather than genes mapped through genomic clones or linked markers). Such data are available for 21 genes out of the 42 that are known to correspond to mutations in Additional file 1. Allele: allele designation; Abbr.: mutant abbreviation; LG: linkage group; Pos.: map position (cM distance from the top of the LG); Gene: gene symbol; Gene Pos.: Median gene position on ZMAP (cM distance from the top of the LG); Panels: mapping panels on which the gene was mapped; Diff.: difference between mutant and gene positions (cM). ^a ^Assignment to LG8 supported by published marker linkages [17]. ^b ^Assignment to LG9 supported by the T51 panel (as shown on the ZFIN website) and by the latest version of the HS map [18].
